# Gasdermin D Inhibits Coronavirus Infection by Promoting the Noncanonical Secretion of Beta Interferon

**DOI:** 10.1128/mbio.03600-21

**Published:** 2022-02-01

**Authors:** Liyuan Zhao, Liang Li, Mei Xue, Xiang Liu, Chengfan Jiang, Wenzhe Wang, Lijie Tang, Li Feng, Pinghuang Liu

**Affiliations:** a State Key Laboratory of Veterinary Biotechnology, Harbin Veterinary Research Institute, Chinese Academy of Agricultural Sciences, Harbin, China; b College of Veterinary Medicine, China Agricultural University, Beijing, China; c College of Veterinary Medicine, Northeast Agricultural University, Harbin, China; University of Calgary

**Keywords:** pyroptosis, GSDMD, coronavirus, TGEV, PDCoV, IFN-β

## Abstract

Pyroptosis, a programmed cell death, functions as an innate immune effector mechanism and plays a crucial role against microbial invasion. Gasdermin D (GSDMD), as the main pyroptosis effector, mediates pyroptosis and promotes releasing proinflammatory molecules into the extracellular environment through pore-forming activity, modifying inflammation and immune responses. While the substantial importance of GSDMD in microbial infection and cancer has been widely investigated, the role of GSDMD in virus infection, including coronaviruses, remains unclear. Enteric coronavirus transmissible gastroenteritis virus (TGEV) and porcine deltacoronavirus (PDCoV) are the major agents for lethal watery diarrhea in neonatal pigs and pose the potential for spillover from pigs to humans. In this study, we found that alphacoronavirus TGEV upregulated and activated GSDMD, resulting in pyroptosis after infection. Furthermore, the fragment of swine GSDMD from amino acids 242 to 279 (242-279 fragment) was required to induce pyroptosis. Notably, GSDMD strongly inhibited both TGEV and PDCoV infection. Mechanistically, the antiviral activity of GSDMD was mediated through promoting the nonclassical release of antiviral beta interferon (IFN-β) and then enhancing the interferon-stimulated gene (ISG) responses. These findings showed that GSDMD dampens coronavirus infection by an uncovered GSDMD-mediated IFN secretion, which may present a novel target of coronavirus antiviral therapeutics.

## INTRODUCTION

Coronaviruses have caused three major pandemics since 2003 and pose serious threats to human and animal health (1–3). Transmissible gastroenteritis virus (TGEV) and porcine deltacoronavirus (PDCoV) are highly contagious enteropathogenic coronaviruses and have been the major causative agents for lethal watery diarrhea in piglets, which results in significant economic loss in the pork industry (2, 4). In addition to being economically important porcine coronaviruses, PDCoV uses the aminopeptidase N (APN) from multiple species, including humans and cats, to enter cells and pose zoonotic potential and potential spillover transmission from pigs to humans (5–7). As severe acute respiratory syndrome coronavirus (SARS-CoV) and SARS-CoV-2 do, TGEV and PDCoV infect both respiratory epithelia and intestinal epithelia, which provide a good model for human highly pathogenic coronaviruses (8, 9). Therefore, a better understanding of the cellular responses following swine enteric coronavirus (SECoV) infection will elucidate the innate immunity of human coronaviruses and identify the novel therapeutic targets for reemerging coronavirus diseases.

Pyroptosis functions as an innate host defense mechanism against intracellular pathogens by eliminating infected cells and thereby curtailing the survival and proliferation of intracellular pathogens (10–12). Gasdermin D (GSDMD), a member of gasdermin family of proteins, was identified as the main executioner of pyroptosis in 2015 (13). GSDMD is a protein composed of an N terminus (N-GSDMD) and C terminus (C-GSDMD) connected by a peptide linker (14–16). Upon activation, the GSDMD linker is cleaved by caspases to separate N-GSDMD from its autoinhibitory C-GSDMD domain (17–19) and form membrane pores through oligomerization, and then excessive membrane pores of N-GSDMD drive a programmed lytic cell death, pyroptosis (20–22). Pyroptosis has been observed in the infection scenario of several coronaviruses, including SARS-CoV-2 (8, 23, 24), murine coronavirus mouse hepatitis virus (MHV) (25), and TGEV (26). GSDMD functioning as the primary effector of canonical pyroptosis is integrated with the host defense against intracellular pathogens (27–30). Recent work shows that GSDMD contributes to the SARS-CoV-2-associated cytokine storm and increases the risk of severe COVID-19 disease (8, 31). However, GSDMD knockout enhances MHV replication in bone marrow-derived macrophages (BMDMs) (25). Whether GSDMD serves protective or detrimental functions in the context of coronavirus infection remains elusive.

Type I IFN (IFN-I) (alpha/beta interferon [IFN-α/β]) is the initial host innate cytokine in response to virus infection and is critical for host defense against virus infection (32–35). IFN-I binds to the ubiquitously expressed IFN-I receptor (IFNAR) through autocrine and paracrine methods and induces a wide range of interferon-stimulated gene (ISG) expression, promoting an antiviral state in bystander cells and restricting viral replication (36–40). Studies from our and other groups have demonstrated that infection with TGEV and PDCoV can induce the transcriptional expression of IFN-I and increase extracellular IFN-β levels (41–43). The plasma membrane pores formed by N-GSDMD also serve as conduits for transporting inflammatory cytokines lacking signal peptide, including interleukin 1β (IL-1β) and IL-18, across intact membrane lipid bilayers and contribute to the host inflammatory responses (31–35). Previous studies also demonstrate that GSDMD plays a crucial role in the unconventional secretion of inflammatory cytokines tumor necrosis factor alpha (TNF-α), IL-6, and CCL2 in macrophages (36). However, the importance of GSDMD in modifying IFN-I production remains elusive. Recent studies demonstrated that GSDMD antagonizes IFN-I production by dampening the cyclic GMP-AMP synthase (cGAS)-dependent signaling pathway in macrophages infected by intracellular bacteria (44, 45). Whether and how GSDMD manipulates IFN-β responses in the context of virus infection are not clear.

Given that GSDMD plays a pivotal role in pyroptosis and the secretion of inflammatory cytokines, we explored the antiviral effect of GSDMD on coronavirus replication in the porcine enteric coronavirus infection model. We found that TGEV infection can upregulate and activate GSDMD. Furthermore, we demonstrated that GSDMD could promote the unconventional pathway secretion of IFN-β, thereby playing an anticoronavirus effect. Our findings highlight the regulatory effect of GSDMD on IFN-β release after coronavirus infection and provide new insights into the antiviral role of GSDMD in the scenario of coronavirus infection.

## RESULTS

### TGEV infection upregulates GSDMD and triggers pyroptosis.

**FIG 1:**
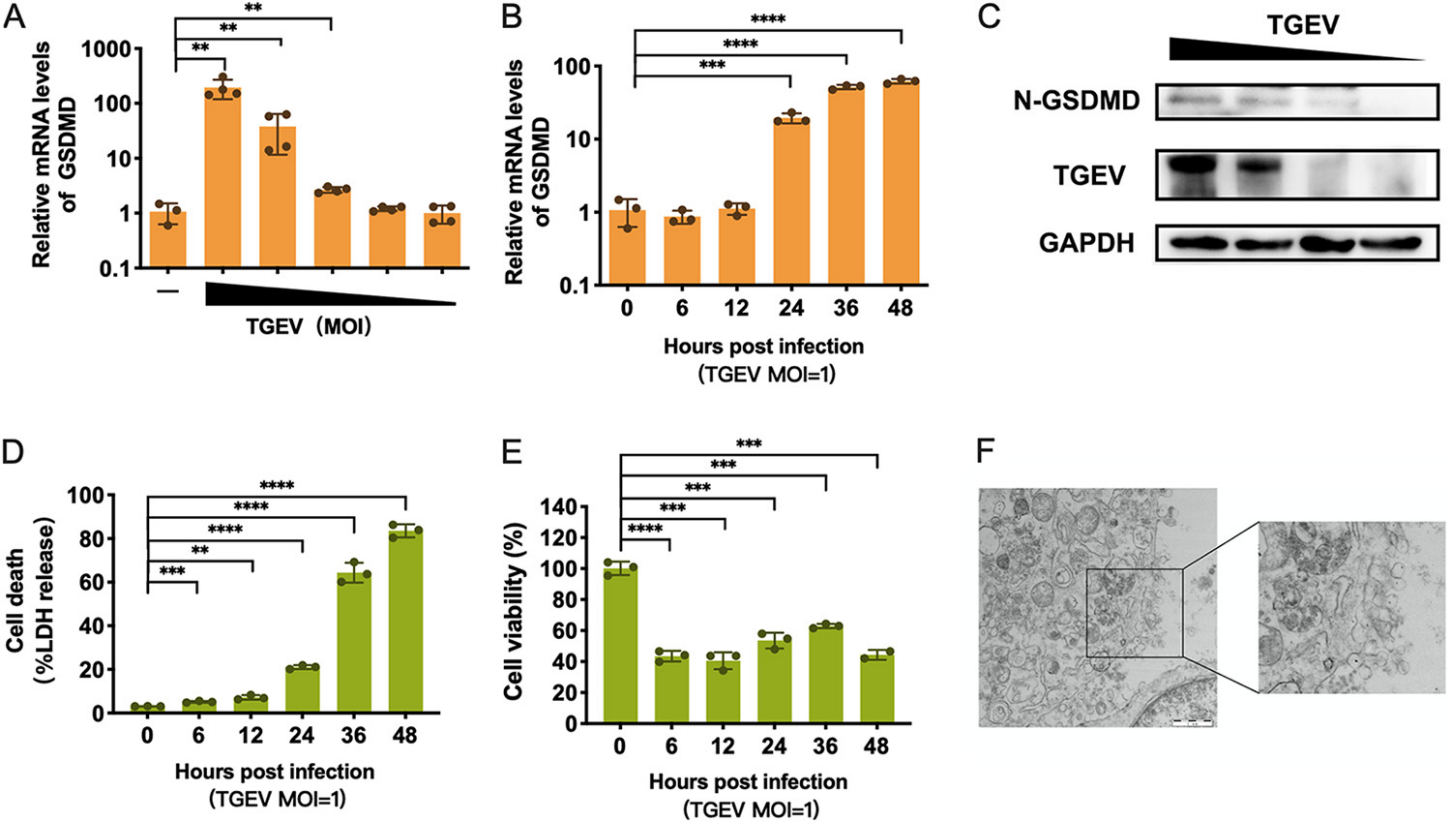
TGEV infection activates GSDMD expression and induces pyroptosis. (A) Dose-dependent upregulated GSDMD mRNA expression was revealed by quantitative real-time PCR in ST cells after TGEV infection at MOIs of 100, 10, 1, 0.1, 0.01, and 0. Total RNA was collected 24 h after infection. (B) Time-dependent upregulated of GSDMD mRNA expression. Total RNA was isolated at 0, 6, 12, 24, 36, and 48 hpi at an MOI of 1 and then evaluated by qPCR. (C) N-GSDMD was activated after TGEV infection. ST cells were infected with TGEV at MOIs of 10, 1, 0.1, and 0; after 24 h of infection, cell lysates were harvested and identified by Western blot analysis. GAPDH was used as an internal loading control. (D and E) Assays for LDH release and cell viability in ST cells at 0 h to 48 h after TGEV infection. (F) Morphological changes of the cell membrane in ST cells after infection with TGEV at 24 h were observed by transmission electron microscopy. The means and SD of the results from three independent experiments are shown. **, *P < *0.01; ***, *P < *0.001; ****, *P < *0.0001.

To determine whether pyroptosis occurs in TGEV infection, we initially monitored the mRNA expression of GSDMD after TGEV infection at different multiplicities of infection (MOIs) in swine testis (ST) cells. TGEV infection significantly upregulated the mRNA levels of GSDMD and exhibited a dose-dependent manner at 24 h postinfection (hpi) (Fig. 1A). The kinetics of GSDMD mRNA in ST cells infected with TGEV at an MOI of 1 showed a substantial upregulation of GSDMD expression from 24 hpi (Fig. 1B). To assess endogenous GSDMD activation after TGEV infection, the cleaved N-GSDMD was detected by Western blotting. The protein levels of N-GSDMD in TGEV-infected ST cells increased and displayed a dose-dependent response to TGEV infection (Fig. 1C), indicating that TGEV infection resulted in the activation of GSDMD. This membrane rupture by the N-GSDMD-formed pores results in the release of cytosolic lactate dehydrogenase (LDH) into the extracellular space, and the presence of extracellular LDH is widely recognized as a marker of pyroptosis (46). LDH release from the TGEV-infected cells was gradually elevated after TGEV infection and coincided with the increased GSDMD expression (Fig. 1B and D). The increased LDH release was consistent with the cell activity after TGEV infection measured by ATP (Fig. 1E). Pyroptosis is characterized by swelling followed by rupture of the plasma membrane, resulting in the release of small cytoplasmic contents, distinct from the other two lytic programmed cell deaths (apoptosis and necroptosis) by their morphology (47). The typical morphological features of pyroptosis, such as rupture of the cell membrane and contents released, were also observed by electron microscopy, suggesting that TGEV infection causes pyroptosis (Fig. 1F). Together, these data show that TGEV infection promotes GSDMD expression and induces the activation of GSDMD, which eventually leads to pyroptosis.

### Amino acids 242 to 279 are required for pyroptosis induced by GSDMD.

**FIG 2:**
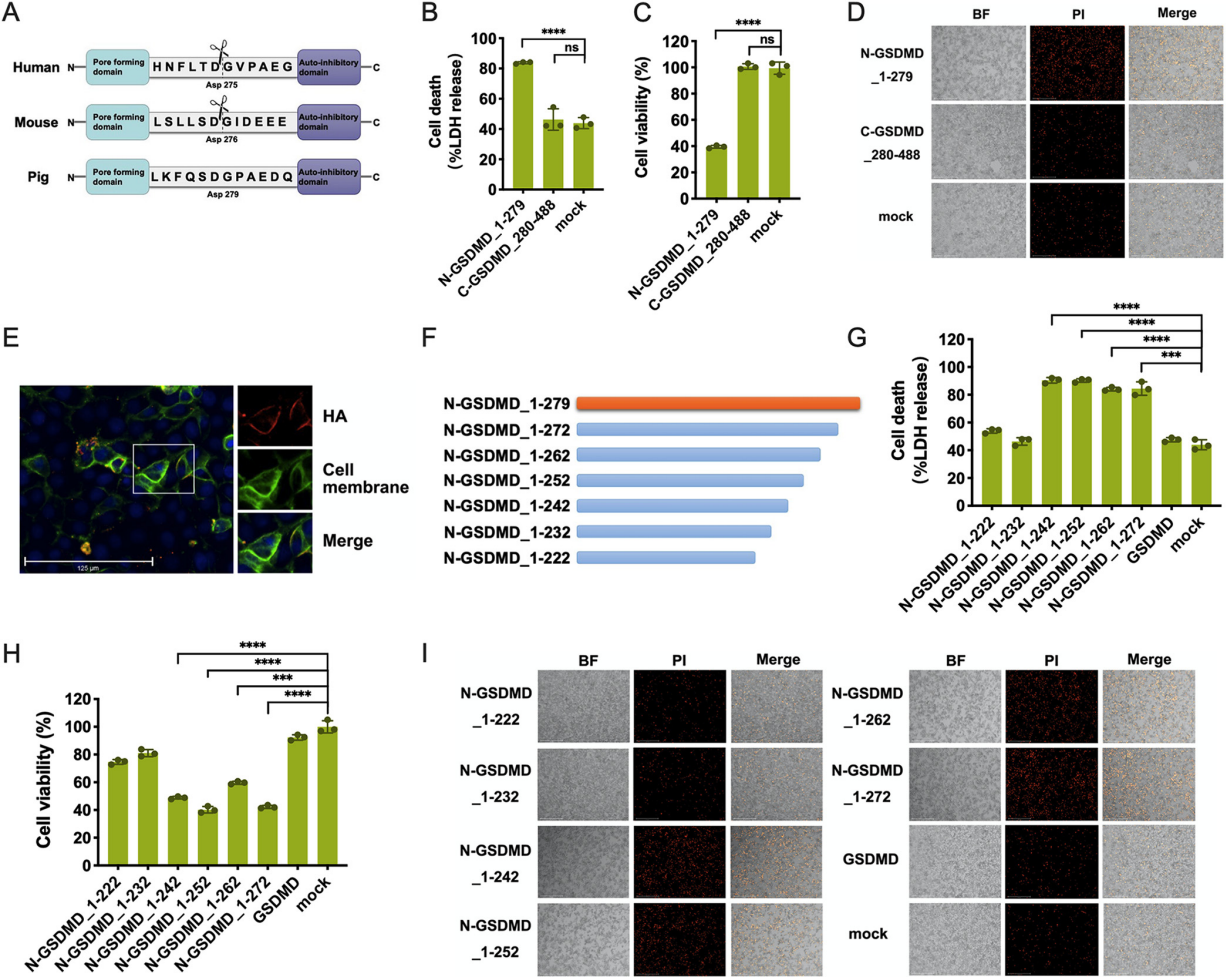
The fragment of GSDMD from amino acids 242 to 279 can induce pyroptosis. (A) Amino acid comparison of caspase-1 cleavage sites in human, murine, and swine GSDMD. (B and C) Assays for LDH release and cell viability after N-GSDMD_1-279 and C-GSDMD_280-488 fragment transfection in 239T cells. (D) N-GSDMD_1-279 caused cell membrane damage. 293T cells were stained with PI after transfection with N-GSDMD_1-279 and C-GSDMD_280-488 for 24 h. (E) N-GSDMD_1-279 was localized in the cell membrane after transfection of ST cells. ST cells were transfected with hemagglutinin (HA)-tagged N-GSDMD_1-279. Twenty-four hours later, HA was labeled with red dye, the cell membrane was dyed green with DIO, and a fluorescence microscope was used to observe the distribution of the N-GSDMD_1-279. (F) Schematic diagrams of deletion mutants of GSDMD. (G and H) Assays in 293T cells transfected with N-GSDMD_1-222, N-GSDMD_1-232, N-GSDMD_1-242, N-GSDMD_1-252, N-GSDMD_1-262, and N-GSDMD_1-272 truncated fragments and full-length GSDMD for LDH release and cell viability. (I) Cell death induced by truncated fragments of GSDMD. 293T cells were stained with PI at 24 h after being transfected with truncated GSDMD fragments and full-length GSDMD. BF, bright field. The means and SD of the results from three independent experiments are shown. ***, *P < *0.001; ****, *P < *0.0001. ns, not significant.

Swine GSDMD, a protein composed of 488 amino acids, contains two defined domains (N-GSDMD and C-GSDMD) separated by a linker region, just like human and mouse GSDMD (Fig. 2A). The GSDMD junction area is cleaved by caspase-1 at the conserved residue D275 in humans (9, 13, 48) and by caspase-4/11 at D276 in mice (27, 49). The cleavage site of swine GSDMD was predicted at D279 based on the alignment with human and murine GSDMD sequences (Fig. 2A). To verify the functional N-terminal fragment of swine GSDMD, we constructed a GSDMD N-terminal fragment from amino acids 1 to 279 (N-GSDMD_1-279) and C-terminal fragment from amino acids 280 to 488 (C-GSDMD_280-488). The transient overexpression of N-GSDMD_1-279 instead of C-GSDMD_280-488 significantly increased LDH release and decreased cell viability, suggesting the induction of pyroptosis by N-terminal GSDMD (Fig. 2B and C), consistent with the result of membrane-impermeant dye propidium iodide (PI) staining that the overexpression of N-terminal fragment 1-279 substantially increased the number of PI-positive cells over the mock controls (Fig. 2D). The cytosolic cleaved N-GSDMD fragment has to translocate to the plasma membrane and bind to phospholipids to form pores in the plasma membrane (46). Next, we determined the localization of N-GSDMD_1-279 in the transient expressing cells. The result showed that N-GSDMD_1-279 was mainly located on the cell membrane, further confirming the ability of swine N-GSDMD_1-279 to form pores in the plasma membrane and induce pyroptosis (Fig. 2E).

To further clarify the required fragments of the GSDMD N terminus to mediate pyroptosis-inducing activity, we next constructed truncated fragments of GSDMD with different lengths as shown in Fig. 2F (N-GSDMD_1-222, N-GSDMD_1-232, N-GSDMD_1-242, N-GSDMD_1-252, N-GSDMD_1-262, and N-GSDMD_1-272). The results showed an increase in LDH release and a decrease of cell activity after transient transfection with the N-GSDMD_1-242, N-GSDMD_1-252, N-GSDMD_1-262, and N-GSDMD_1-272 (Fig. 2G and 2H), accompanied by a substantial increase in the number of PI-staining cells (Fig. 2I). However, N-GSDMD_1-222, N-GSDMD_1-232, and full-length GSDMD did not induce significant cell death. These results suggest that the region of GSDMD from amino acids 242 to 279 is critical for GSDMD pore-forming activity.

### GSDMD inhibits the replication of TGEV.

**FIG 3:**
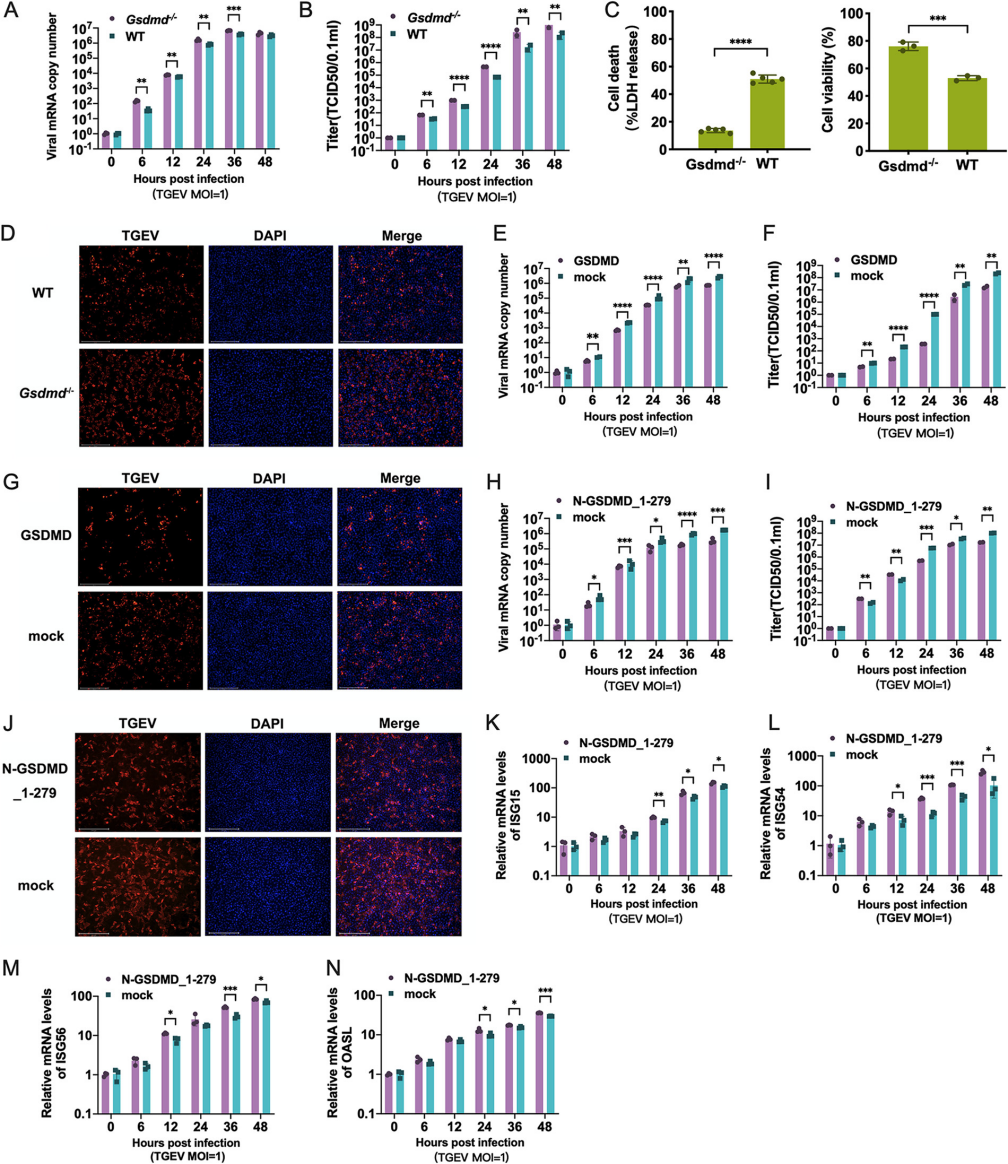
GSDMD can inhibit TGEV replication. (A) Relative mRNA levels of TGEV in *Gsdmd*^−/−^ ST cells and WT ST cells at different time points after infection. Total RNA was extracted at 0, 6, 12, 24, 36, and 48 hpi and then evaluated by quantitative real-time PCR using SYBR green. (B) One-step growth curve of TGEV in *Gsdmd*^−/−^ ST cells and WT ST cells. (C) Attenuation of pyroptosis caused by TGEV in *Gsdmd*^−/−^ ST cells. Assays for LDH release and cell viability were done after TGEV infection in *Gsdmd*^−/−^ ST cells and WT ST cells. (D) *Gsdmd* deletion increased TGEV replication. *Gsdmd*^−/−^ ST cells and WT ST cells were infected with TGEV at an MOI of 1. After 24 h, the cells were fixed with 4% paraformaldehyde and observed for infection of TGEV under a fluorescence microscope by staining the nucleoprotein of TGEV. (E to G) Overexpression of GSDMD inhibited TGEV replication. ST cells were transfected with GSDMD plasmid; empty vector was used as a control. After 24 h of transfection, cells were infected with TGEV at an MOI of 1. The relative mRNA levels (E) and one-step growth curve (F) of TGEV after GSDMD overexpression were measured. (G) TGEV infection was observed by fluorescence staining after 24 h of infection. (H to J) N-GSDMD_1-279 inhibits TGEV replication. ST cells were infected with TGEV at an MOI of 1 after transfection with N-GSDMD_1-279 for 24 h, and an empty vector was used as a control. The relative mRNA levels (H) and one-step growth curve (I) of TGEV after the N-GSDMD_1-279 transfected were measured. (J) The infection was observed by N protein staining of TGEV at 24 hpi. (K to N) The expression levels of ISG15, ISG54, ISG56, and OASL in the N-GSDMD1-279 transfected ST cells were analyzed after TGEV infection. The means and SD of the results from three independent experiments are shown. *, *P < *0.05; **, *P < *0.01; ***, *P < *0.001; ****, *P < *0.0001.

Next, we explored whether GSDMD affects the replication of TGEV. To this end, we initially constructed a *Gsdmd* knockout (*Gsdmd*^−/−^) ST cell line by CRISPR-Cas9. The *Gsdmd* knockout was confirmed by sequencing and protein Western blotting (data not shown). Compared with that of wild-type (WT) ST cells, TGEV infection was largely increased in the *Gsdmd*^−/−^ ST cells as measured by the TGEV replication kinetics curve of viral genomes (Fig. 3A) and infectious particles (Fig. 3B). The increased TGEV infection in the *Gsdmd*^−/−^ ST cells relative to wild-type ST cells was observed starting at 6 hpi (Fig. 3A and B), indicating that GSDMD manipulates TGEV infection not through affecting the viral entry. As expected, the pyroptosis of TGEV-infected *Gsdmd*^−/−^ ST cells measured by LDH release was significantly reduced compared with that of wild-type ST cells, while the viability of *Gsdmd*^−/−^ ST cells increased (Fig. 3C). The enhanced TGEV infection was confirmed by TGEV nucleocapsid (N) protein indirect immunofluorescence (IFA) (Fig. 3D), which was in line with the result of transient overexpression of GSDMD, i.e., that the replication of TGEV was significantly inhibited in ST cells transfected with GSDMD vector compared with mock vector control as measured by the quantification of viral RNA, virus titration, and N protein IFA (Fig. 3E to G, respectively). These results demonstrated that GSDMD dampens TGEV infection.

The known primary function of GSDMD is to form pores in the plasma membrane by N-GSDMD after activation, which contributes to the nonlytic release of inflammatory cytokines and pyroptosis depending on the degree and timing of GSDMD pore-forming activity (28–30). To clarify the underlying mechanisms of the anti-TGEV activity of GSDMD, we initially assessed the effect of N-GSDMD_1-279 with pore-forming activity on TGEV infection. The overexpression of N-GSDMD_1-279 reduced the replication of TGEV as measured by TGEV replication kinetics quantified by viral RNA and titers (Fig. 3H and I, respectively). The suppression of TGEV infection by N-GSDMD_1-279 was confirmed by TGEV N protein IFA (Fig. 3J). Together, these results demonstrated that GSDMD inhibits the replication of TGEV through GSDMD pore activity.

### GSDMD promotes RNA-elicited IFN-β release.

Next, we further explored the underlying mechanisms through which GSDMD suppresses TGEV infection. The results of TGEV replication kinetics in *Gsdmd* knockout or overexpression ST cells showed that GSDMD-mediated viral suppression occurred starting at 6 hpi and was not observed at 0 h, indicating that GSDMD does not affect TGEV entry. Given the critical roles of the IFN-elicited ISG response against virus infection, we hypothesized that GSDMD suppresses TGEV infection by enhancing the IFN-mediated antiviral ISG responses induced by TGEV infection. We initially monitored the expression of ISGs in TGEV-infected ST cells with N-GSDMD_1-279 transient expression after TGEV infection. As we expected, unlike the TGEV infection result that the overexpression of N-GSDMD_1-279 reduced TGEV infection, the overexpression of N-GSDMD_1-279 promoted the transcriptional mRNA expression of ISG15, ISG54, ISG56, and OASL in ST cells after TGEV infection compared with the mock vector control (Fig. 3K to N). These indicate that GSDMD enhances the IFN response in TGEV-infected ST cells.

**FIG 4:**
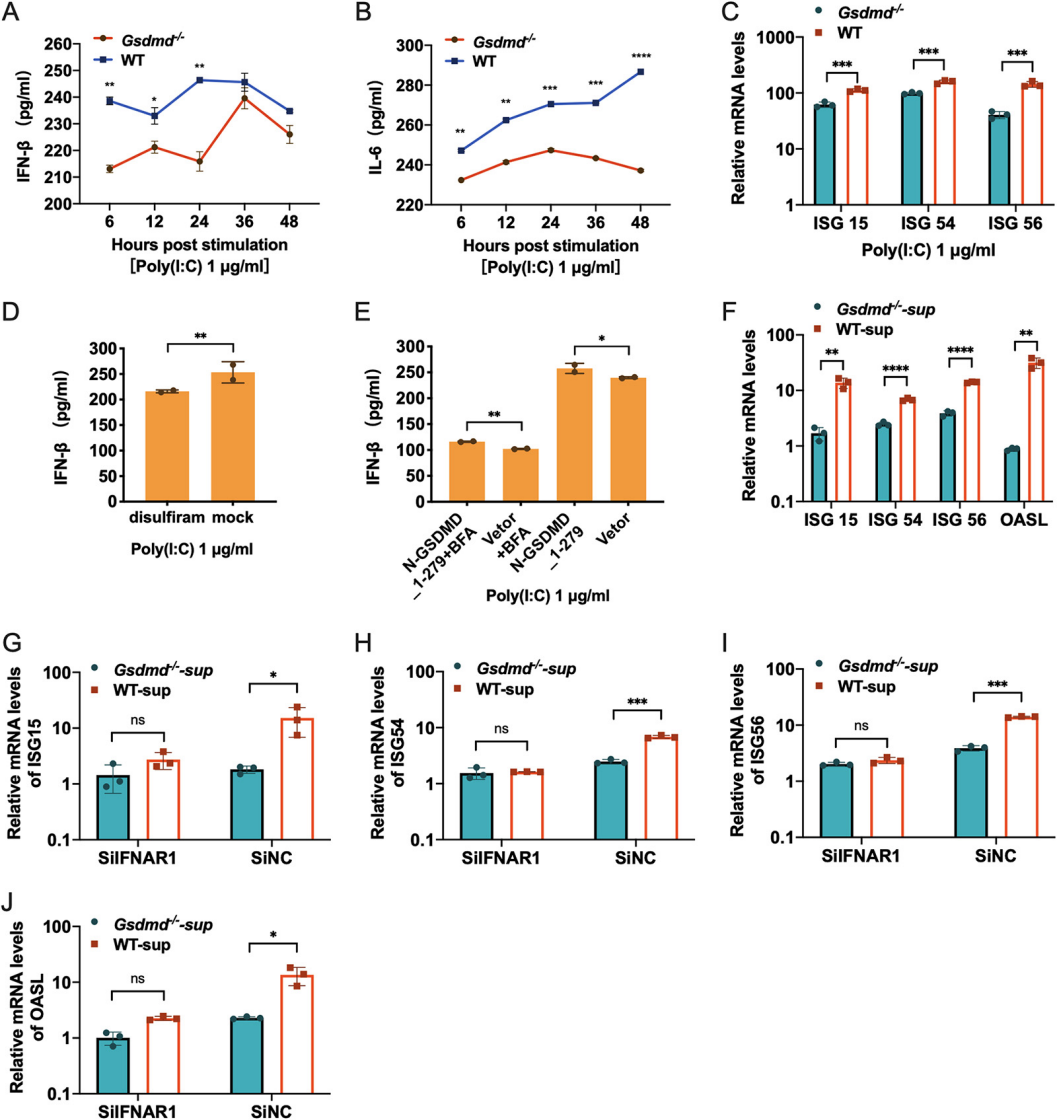
GSDMD deletion reduces IFN-β released by poly(I·C) stimulation. (A) The release of IFN-β was decreased in *Gsdmd*^−/−^ ST cells. The protein levels of supernatant IFN-β in *Gsdmd*^−/−^ ST cells and WT ST cells were analyzed by ELISA at different time points after poly(I·C) stimulation. (B) The protein levels of IL-6 were measured in *Gsdmd*^−/−^ ST cells and WT ST cells after poly(I·C) stimulation. (C) The expression of ISG15, ISG54, and ISG56 was downregulated in *Gsdmd*^−/−^ ST cells. *Gsdmd*^−/−^ ST cells and wild-type ST cells were stimulated with poly(I·C), total RNA was collected at 36 h after stimulation, and then the mRNA levels of ISG15, ISG54, and ISG56 were measured. (D) ST cells were pretreated with disulfiram 1 h before poly(I·C) stimulation, and the supernatants were collected for IFN-β protein determination after 24 h. (E) ST cells were transfected with N-GSDMD_1-279 for 24 h, and the cells were transfected with 250 ng of poly(I·C). BFA was added 1 h before poly(I·C) stimulation, and the final concentration was maintained at 3 μg/mL. The supernatants were collected after 24 h, and the protein level of IFN-β was measured by ELISA. (F) *Gsdmd*^−/−^ ST cells and wild-type ST cells were transfected with 250 ng of poly(I·C), and then the medium was changed to DMEM 6 h after transfection, and cell supernatants were collected after 24 h. The collected cell supernatants were added to unstimulated ST cells; after 24 h, RNA was extracted for qPCR determination of ISG15, ISG54, ISG56, and OASL. (G to J) ST cells were transfected with IFNAR1 siRNA or control siRNA for 24 h, and the cell supernatants collected for panel F were added. After 24 h, the total RNA was extracted, and the expression of ISG15, ISG54, ISG56, and OASL was measured by qPCR. The means and SD of the results from three independent experiments are shown. *, *P < *0.05; **, *P < *0.01; ***, *P < *0.001; ****, *P < *0.0001.

Previous studies showed that TGEV infection strongly elicits IFN-β production (50). IFN-I can function through paracrine forms to exert antiviral activity in bystander cells (51, 52). Several studies have found that the N-GSDMD-formed pores in the plasma membrane result in the release of small cytosolic contents, including inflammatory cytokines (53–55). We assumed that the pores formed by N-GSDMD could also promote cytosolic IFN-β release into extracellular space to suppress TGEV infection. To test this, we initially monitored IFN-β production in *Gsdmd*^−/−^ ST cells and WT ST cells after stimulation with poly(I·C), an analog of double-stranded RNA that can robustly induce IFN-I. Surprisingly, the supernatant levels of IFN-β protein notably showed a substantial decrease in *Gsdmd*^−/−^ ST cells within 48 h after poly(I·C) stimulation compared with WT ST cell supernatant (Fig. 4A). Furthermore, the decreased supernatant IFN-β by poly(I·C)-primed *Gsdmd*^−/−^ ST cells was sustained throughout the study period. Similarly, consistent with previous studies showing that GSDMD was critical for IL-6 nonclassical secretion (44), the decreased supernatant IL-6 protein in response to poly(I·C) stimulation was also observed in *Gsdmd*^−/−^ ST cells compared with WT ST cells (Fig. 4B). Consistent with supernatant IFN-β protein levels, reduced ISG15, ISG54, and ISG56 induction were observed in *Gsdmd*^−/−^ ST cells compared with WT ST cells after poly(I·C) treatment (Fig. 4C).

To confirm the enhanced IFN-β release by GSDMD pore-formed activity, we monitored the supernatant IFN-β in ST cells following poly(I·C) stimulation in the presence of disulfiram, an inhibitor of pore formation by GSDMD. Treatment of cells with disulfiram reduced the release of IFN-β induced by poly(I·C) (Fig. 4D), suggesting that the pore-forming effect of GSDMD is involved in the IFN release. To further verify the GSDMD-mediated noncanonical secretion of IFN, we monitored the supernatant IFN-β of ST cells transfected with N-GSDMD_1-279 after stimulation with poly(I·C) in the presence of brefeldin A (BFA), a specific inhibitor of cytokine classical secretion pathway. We found that BFA significantly reduced the poly(I·C)-induced canonical IFN-β release but did not abolish the enhanced supernatant IFN-β by the N-GSDMD_1-279 overexpression (Fig. 4E), indicating that N-GSDMD_1-279 promotes IFN-β release via a nonclassical pathway. Moreover, we measured the ability of the secreted supernatant IFN-I of *Gsdmd*^−/−^ ST cells or WT ST cells harvested at 24 h after poly(I·C) stimulation to induce ISG in ST cells. Compared with the supernatants of WT ST cells, the supernatants of *Gsdmd*^−/−^ ST cells induced significantly lower expression levels of ISG15, ISG54, ISG56, and OASL in ST cells (Fig. 4F), indicating less IFN-I present in the supernatants of *Gsdmd*^−/−^ ST cells. To further confirm whether the varied concentration of supernatant IFN-I specifically mediates the different ability to elicit ISGs, we stimulated ST cells with the supernatants after knocking down the receptor of IFN-I (IFNAR1) by small interfering RNA (siRNA). Knockdown of IFNAR1 receptor by specific siRNA significantly alleviated the upregulation of ISGs induced by the supernatants and abolished the different ability of *Gsdmd*^−/−^ ST cells or WT ST cells supernatants to induce ISG15, ISG54, ISG56, and OASL expression (Fig. 4G to J). Together, these data demonstrate that GSDMD enhances IFN-I production by strongly promoting the release of IFN-I, although GSDMD reins in poly(I·C)-elicited IFN-β transcription.

### GSDMD inhibits TGEV replication by promoting IFN-β release.

**FIG 5:**
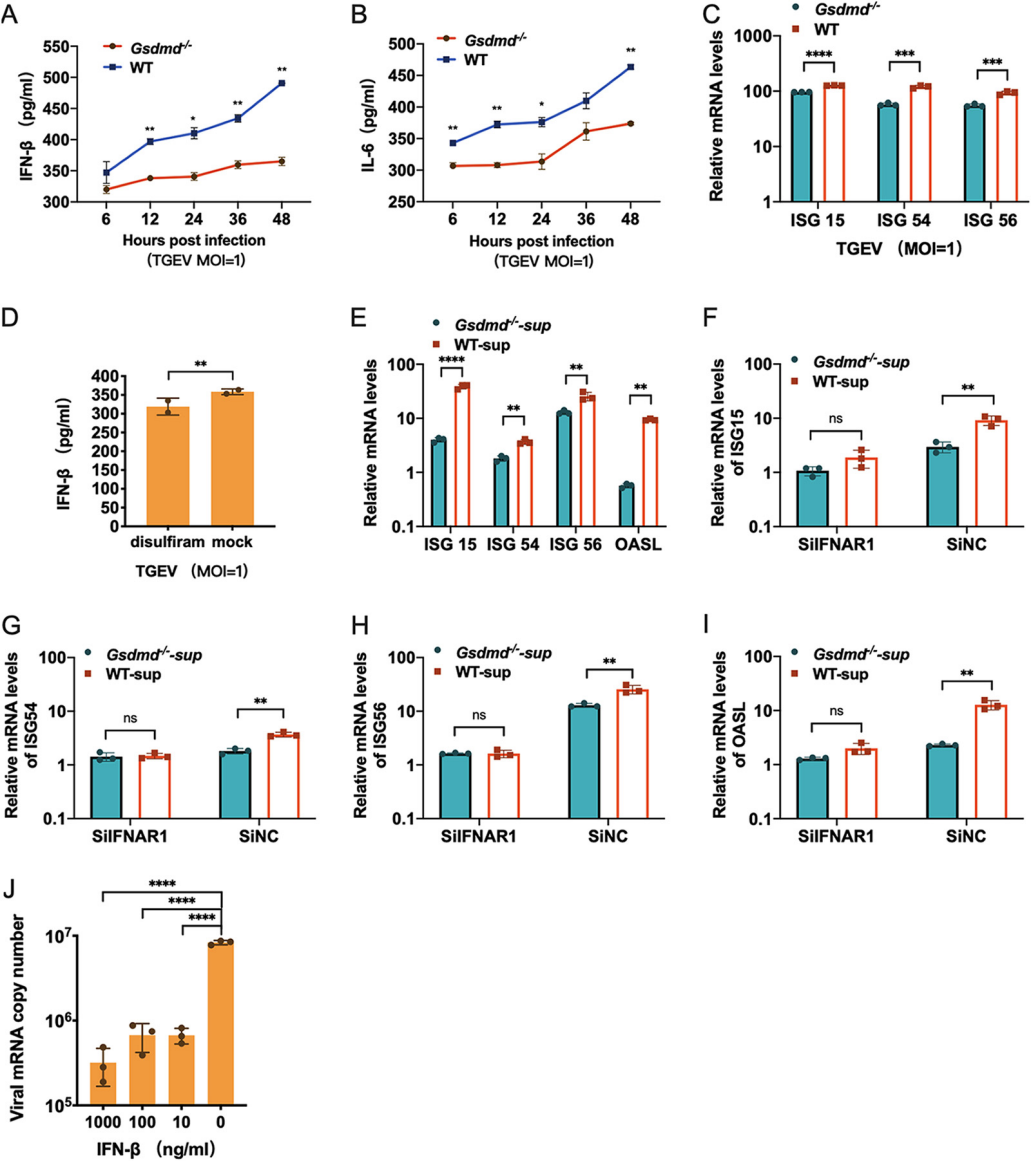
GSDMD inhibits TGEV replication by regulating IFN release. (A and B) The release of IFN-β and IL-6 was reduced in *Gsdmd*^−/−^ ST cells after infection with TGEV. *Gsdmd*^−/−^ ST cells and wild-type ST cells were infected with TGEV, and then IFN-β and IL-6 protein levels of supernatant were determined by ELISA. (C) *Gsdmd*^−/−^ ST cells induce lower ISG expression after TGEV infection. *Gsdmd*^−/−^ ST cells and wild-type ST cells were infected with TGEV, and total RNA was collected at 36 hpi. Relative mRNA levels of ISG15, ISG54, and ISG56 were measured using qPCR. (D) ST cells were pretreated with disulfiram 1 h before TGEV infection, and the supernatants were collected for IFN-β determination at 24 hpi. (E) *Gsdmd*^−/−^ ST cells and wild-type ST cells were infected with TGEV at an MOI of 1, and cell supernatants were collected at 24 hpi. All supernatants were irradiated with UV for 2 h to ensure that TGEV was inactivated. Uninfected ST cells were treated with the inactivated supernatants for 24 h, and the relative mRNA levels of ISG15, ISG54, ISG56, and OASL were measured. (F to I) ST cells were transfected with IFNAR1 siRNA or control siRNA for 24 h, and the inactivated supernatants obtained before were added. After 24 h, total RNA was isolated, and the relative mRNA expression of ISG15, ISG54, ISG56, and OASL was measured by qPCR. (J) IFN-β suppressed TGEV replication in a dose-dependent manner. Viral replication was measured after treatment with porcine IFN-β. The relative mRNA levels of TGEV are presented. The means and SD of the results from three independent experiments are shown. *, *P < *0.05; **, *P < *0.01; ***, *P < *0.001; ****, *P < *0.0001.

We demonstrated above that GSDMD could promote IFN/ISG responses by increasing the release of IFN-β after poly(I·C) poly(I·C)stimulation. Therefore, it is rational to hypothesize that GSDMD dampens TGEV infection by enhancing the IFN-I-mediated ISG response through paracrine forms. We initially explored whether GSDMD could regulate the IFN-β release in the context of TGEV infection. Compared with the findings with WT ST cells, *Gsdmd* knockout substantially reduced the protein levels of supernatant IFN-β after TGEV infection (Fig. 5A). As expected, *Gsdmd* knockout undermined the secretion of TGEV-induced IL-6 (Fig. 5B). Concurrently, the induction of ISGs in TGEV-infected *Gsdmd*^−/−^ ST cells was also less than that of WT ST cells with the same dose of TGEV (Fig. 5C). Inhibition of GSDMD pore formation by disulfiram reduced the supernatant IFN-β induced by TGEV (Fig. 5D). Consistent with the supernatant IFN-β results, the supernatant of TGEV-infected WT ST cells induced higher ISG15, ISG54, ISG56, and OASL expression in the uninfected ST cells than the supernatants of TGEV-infected *Gsdmd*^−/−^ ST cells (Fig. 5E). Furthermore, IFNAR1 knockdown of ST cells relieved the discrepant abilities between the supernatants of TGEV-infected *Gsdmd*^−/−^ ST cells and WT ST cells to elicit ISG15, ISG54, ISG56, and OASL expression in ST cells, indicating that various concentrations of IFN-I largely account for the different capacities of the supernatants to induce ISGs (Fig. 5F to I). Furthermore, exogenous IFN-β pretreatment robustly inhibited TGEV infection in ST cells, suggesting that TGEV is sensitive to IFN-β antiviral activity (Fig. 5J). Together, these results show that GSDMD inhibits TGEV infection by promoting IFN-β release.

### GSDMD inhibits PDCoV infection by promoting IFN-β release.

**FIG 6:**
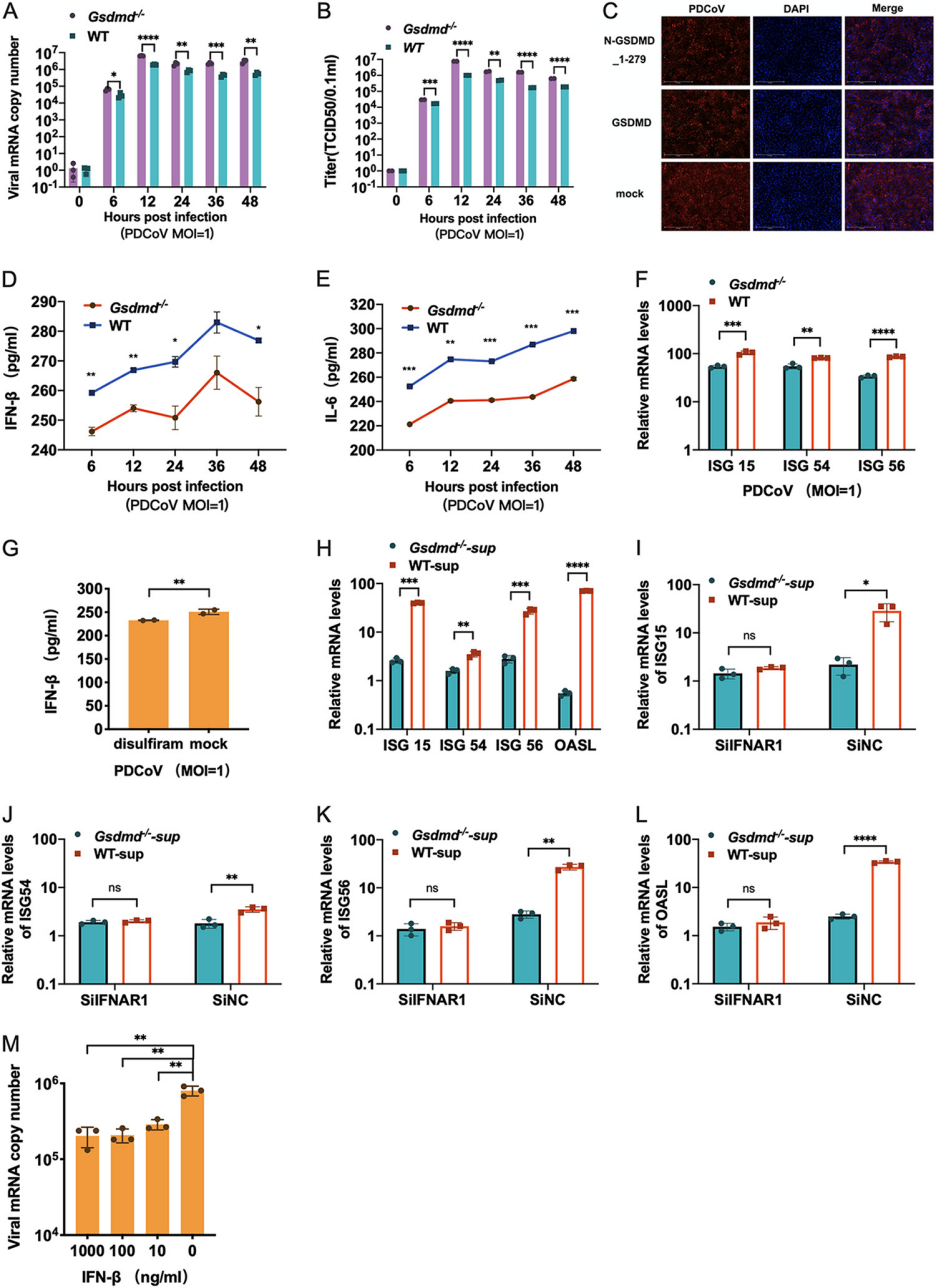
GSDMD regulates the release of IFN-β during PDCoV infection and inhibits its replication. (A and B) *Gsdmd* deletion increased PDCoV replication. The relative mRNA levels (A) and one-step growth curve (B) of PDCoV in *Gsdmd*^−/−^ ST cells and WT ST cells at different time points after infection are shown. (C) Full-length GSDMD and N-GSDMD_1-279 inhibited PDCoV replication. ST cells were transfected with full-length GSDMD plasmid and N-GSDMD_1-279 plasmid, and empty vector was used as a control. After 24 h of transfection, cells were infected with PDCoV at an MOI of 1 and then observed for PDCoV infection by fluorescence staining at 24 hpi. (D and E) PDCoV infection in *Gsdmd*^−/−^ ST cells induced less release of IFN-β and IL-6. IFN-β and IL-6 levels in the supernatant were determined by ELISA after PDCoV infection. (F) Relative mRNA levels of ISG15, ISG54, and ISG56. (G) ST cells were pretreated with disulfiram 1 h before PDCoV infection, and the supernatants were collected for IFN-β determination at 24 hpi. (H) Relative mRNA levels of ISG15, ISG54, ISG56, and OASL of supernatants treated or IFNAR1 knockdown treated (I to L). The experimental phases are indicated as above for Fig. 5F to I. (M) IFN-β inhibited PDCoV in a dose-dependent manner. Cells were treated with porcine IFN-β before PDCoV infection. The mRNA levels of PDCoV were measured. The means and SD of the results from three independent experiments are shown. *, *P < *0.05; **, *P < *0.01; ***, *P < *0.001; ****, *P < *0.0001.

We and others previously showed that PDCoV infection induces evident IFN-I production, like TGEV infection (43, 56). We next explored whether GSDMD undermines PDCoV infection by enhancing the release of IFN-I. PDCoV growth kinetics showed that the *Gsdmd* knockout promoted PDCoV infection as quantified by viral genomes (Fig. 6A) and titers (Fig. 6B), indicating that GSDMD suppresses PDCoV infection. Moreover, the inhibition of PDCoV infection by GSDMD was confirmed by the transient overexpression of functional N-GSDMD_1-279 and full-length GSDMD measured by PDCoV S protein IFA (Fig. 6C). In addition, the *Gsdmd* knockout resulted in a decreased level of supernatant IFN-β and IL-6 protein after PDCoV infection (Fig. 6D and E) and a reduced level of antiviral ISG induction in the PDCoV-infected *Gsdmd*^−/−^ ST cells compared with the infected WT ST cells (Fig. 6F). Meanwhile, the release of IFN-β was inhibited by disulfiram after PDCoV infection (Fig. 6G), suggesting that partial PDCoV-elicited IFN-β is released through GSDMD-formed pores. The reduced supernatant IFN-β in the PDCoV-infected *Gsdmd*^−/−^ ST cells compared with WT ST cells was further confirmed by measuring the ISG (ISG15, ISG54, ISG56, and OASL) induction in the uninfected ST cells after stimulating the supernatant cells harvested from PDCoV-infected *Gsdmd*^−/−^ ST or WT ST cells (Fig. 6H). Moreover, IFNAR1 silencing essentially reduced the ISG expression elicited by the supernatants harvested from the PDCoV-infected *Gsdmd*^−/−^ ST cells and abolished the supernatant’ ISG induction disparity between PDCoV-infected *Gsdmd*^−/−^ ST and WT ST cells (Fig. 6I to L). Like TGEV, PDCoV was sensitive to the antiviral activity of IFN-β and displayed a dose-dependent manner regarding IFN-β inhibition (Fig. 6M). Collectively, these data indicate that GSDMD also inhibits PDCoV infection by promoting the release of IFN-β.

## DISCUSSION

Pyroptosis functions as an innate immune effector mechanism by removing the replicative niche and enhancing the release of inflammatory factors or other active molecules (57–59). Here, we show that alphacoronavirus TGEV infection activated GSDMD and induced pyroptosis. Pyroptosis and cleavage of GSDMD induced by TGEV infection have been recently reported by another group (26). Pyroptosis and GSDMD activation are observed in the context of infection with other coronaviruses, such as SARS-CoV-2 and MHV (25, 31, 57, 60). SARS-CoV-2 infection results in GSDMD cleavage and triggers pyroptosis via inflammasome caspase-1 in human monocytes *in vitro* and *in vivo* (31). It seems that it is a common phenomenon for coronaviruses to induce pyroptosis. However, the importance of coronavirus-induced pyroptosis in manipulating virus infection itself is poorly investigated.

The essential roles of GSDMD in intracellular bacterial infection have been widely recognized. It is unknown whether GSDMD serves protective or detrimental functions for virus infection, including coronavirus infection. We demonstrate that GSDMD substantially inhibited infection by swine enteric coronaviruses TGEV and PDCoV by promoting the release of IFN-β and enhancing antiviral ISG responses (Fig. 5 and 6). The detrimental role of GSDMD in TGEV and PDCoV infection is in line with another recent work showing that inhibition of NLRP3-mediated pyroptosis enhanced the replication of TGEV in intestinal epithelial cells (26). These findings are consistent with the MHV study showing that GSDMD deficiency increased cell death after MHV infection, though the authors did not explore what impact GSDMD deficiency has on MHV infection (25). The viral inhibition of GSDMD was also reported in the murine rotavirus intestinal infection model (61).

Furthermore, we demonstrate that the pore-formed activity of GSDMD is associated with the antiviral activity of GSDMD (Fig. 6), which is why viruses have also evolved multiple mechanisms to avoid the activation of GSDMD. Ma et al. recently showed that SARS-CoV-2 blocks GSDMD cleavage by binding SARS-CoV-2 N protein to the GSDMD linker region (67). Enterovirus 71 protease 3C can counteract GSDMD function by cleaving GSDMD at Q193-G194 in the N terminus to produce a nonfunctional N-terminal fragment (62). There is a pressing need to explore how TGEV and PDCoV counteract GSDMD antiviral activity.

Our data suggest that pores formed by GSDMD represent an important supplemental mechanism of IFN-I secretion in the context of coronavirus infection, which provides an unknown mechanism by GSDMD to curtail coronavirus infection. Previous studies demonstrated that GSDMD plays a critical role in releasing inflammatory cytokines, including IL-1β, IL-18, and IL-6, in lytic pyroptotic and nonlytic intact cells. No bioactive IL-1β is released from *Gsdmd* knockout macrophages since proIL-1β lacks a signal peptide that directs them to the secretory pathway (63). Unlike IL-1β and IL-18, both IFN-β and IL-6 have a signal peptide and are secreted via the constitutive (or continuous) secretory pathway after synthesis (64–66). Consistent with this, we observed that IFN-β protein was present in the supernatant of TGEV-infected *Gsdmd*^−/−^ ST cells during the study period even though the supernatant IFN-β concentration was greatly lower than for WT ST cell supernatant, indicating that GSDMD pores provide an important supplemental pathway for IFN-β release except for the constitutive secretory pathway of IFN-β. The enhanced IFN-β release by GSDMD seems a common scenario in RNA virus infection since it was observed in poly(I·C) stimulation and PDCoV infection, in contrast to the study of GSDMD in modifying microbial DNA-induced IFN-I production during bacterial infection showing that GSDMD dampens cGAS-dependent IFN-I elicitation by promoting intracellular K^+^ efflux (45). We also observed increased IFN-β mRNA transcription in *Gsdmd*^−/−^ ST cells in response to poly(I·C) stimulation compared with that in WT ST cells, indicating that GSDMD dampens the transcription of IFN-β (data not shown), which further emphasizes the importance of GSDMD in releasing IFN-β in the context of RNA virus infection, given that GSDMD knockout resulted in more cytosolic IFN-β production in response to poly(I·C) stimulation.

Therefore, our data revealed an undiscovered mechanism employed by the host to resist coronavirus through the GSDMD-mediated modification of the IFN response. However, we recognize our study's limitations. We did not clarify that GSDMD-mediated IFN-β release is through lytic pyroptosis or the pores in the intact plasma membrane of living cells formed by N-GSDMD. Further investigations of the roles of GSDMD in releasing the IFN-I in various cells and virus infection will help us better understand the host’s finely tuned IFN-I response in the context of virus infection.

## MATERIALS AND METHODS

### Cell culture and virus infection.

Swine testis (ST) cells and HEK-293T cells were cultured in Dulbecco’s modified Eagle’s medium (DMEM; Gibco) supplemented with 10% heated-inactivated fetal bovine serum (FBS; Gibco). ST cells were infected with TGEV strain H16 (GenBank accession no. FJ755618) (MOI = 1) or mock infected with DMEM. After 2 h of infection at 37°C, the cells were washed three times with DMEM. Then cells were cultured in maintenance medium (DMEM supplemented with 0.3% trypsin and 1% DMSO) at 37°C. The PDCoV strain NH (GenBank accession no. KU981062.1) was used to infect ST cells at an MOI of 1. After incubation for 2 h at 37°C, cells were washed three times to remove the unbound virus and cultured in maintenance medium (DMEM supplemented with 0.4% trypsin) at 37°C.

### Plasmids and antibodies.

Plasmids were constructed by homologous recombination using the ClonExpress Ultra one-step cloning kit (Vazyme, China, Nanjing). Pig full-length GSDMD was cloned from ST cell cDNAs, tagged with hemagglutinin (HA) at the N terminus, and then inserted into a pCAGGS vector. The deletion mutants of GSDMD (N-GSDMD_1-222, N-GSDMD_1-232, N-GSDMD_1-242, N-GSDMD_1-252, N-GSDMD_1-262, N-GSDMD_1-272, N-GSDMD_1-279, and C-GSDMD_280-488) were constructed based on the full-length GSDMD plasmid. The sequences of the primers used are listed in Table 1. All plasmids have been confirmed by nucleotide sequence analysis and verified expression on 293T cells.

**TABLE 1:** Sequences of primers for PCR

Primer	Sequence (5′–3′)
GSDMD-F	CGGAATTCGCATCAGCCTTTGAGAGG
GSDMD-R	CGGGTACCCTAGCAGAGCTGGCTGAGC
1-GSDMD-F	TTCCAGATTACGCTGAATTCATGGCATCAGCCTTTGAGAGG
1-222-R	GGCATGCCCGGGTACCATCACCAGCTGGGCCACC
1-232-R	GGCATGCCCGGGTACCGGGAAGAGAAGGATGTCCCA
1-242-R	GGCATGCCCGGGTACCAGCGGCCTGAAGGTTCG
1-252-R	GGCATGCCCGGGTACCACCGTGGGAGGCGCTATG
1-262-R	GGCATGCCCGGGTACCAGGCGGGAGAACTGCGG
1-272-R	GGCATGCCCGGGTACCTGCTCAGAGGGGAAGCTCAT
1-279-R	GGCATGCCCGGGTACCGTCTGACTGGAACTTCAGGTG
280-488-F	TTCCAGATTACGCTGAATTCGGGCCCGCGGAGGA
280-488-R	CTCCTCCGCGGGCCCCTAGCAGAGCTGGCTGAG

Antibodies against HA were purchased from Abcam (Cambridge, MA), GSDMDC1 antibody (sc-81868) was purchased from Santa Cruz Biotechnology (Dallas, TX), glyceraldehyde-3-phosphate dehydrogenase (GAPDH) mouse monoclonal antibody was purchased from Beyotime (Shanghai, China), and monoclonal antibodies against TGEV N protein and monoclonal antibodies against PDCoV S protein were prepared and stocked by our team.

### Real-time quantitative RT-PCR.

ST cells were infected with TGEV or PDCoV at 6, 12, 24, 36, and 48 h, and the total RNA was extracted using an RNeasy kit (Qiagen Sciences, Hilden, Germany). cDNA was obtained using a PrimeScript II 1st-strand cDNA synthesis kit (TaKaRa, Dalian, China). Quantitative PCR (qPCR) was performed using a LightCycler 480 reverse transcription PCR (RT-PCR) machine (Roche) with SYBR. All primers are listed in Table 2, and all data were analyzed based on the cycle threshold (ΔΔ*C_T_*) method, and GAPDH was used as the internal control.

**TABLE 2:** Real-time qPCR primers

Primer	Sequence (5′–3′)
TGEV-qPCR-F	GCTTGATGAATTGAGTGCTGATG
TGEV-qPCR-R	CCTAACCTCGGCTTGTCTGG
PDCoV-qPCR-F	AGCAACCACTCGTGTTACTTG
PDCoV-qPCR-R	CAACTCTGAAACCTTGAGCTG
ISG54-qPCR-F	CTGGTCACCTGGGGAAACTA
ISG54-qPCR-R	CACCCTTCCTCACAGTCCAT
ISG56-qPCR-F	TCAGAGGTGAGAAGGCTGGT
ISG56-qPCR-R	GCTTCCTGCAAGTGTCCTTC
ISG15-qPCR-F	AGCATGGTCCTGTTGATGGTG
ISG15-qPCR-R	CAGAAATGGTCAGCTTGCACG
OASL-qPCR-F	TCCCTGGGAAGAATGTGCAG
OASL-qPCR-R	CCCTGGCAAGAGCATAGTGT
GSDMD-qPCR-F	ATGGCATCAGCCTTTG
GSDMD-qPCR-R	CTAGCAGAGCTGGCTG
GAPDH-qPCR-F	CCTTCCGTGTCCCTACTGCCAAC
GAPDH-qPCR-R	GACGCCTGCTTCACCACCTTCT

### Chemical treatments.

Poly(I·C) was purchased from Sigma (USA) and used to transfect ST cells at a final concentration of 1 μg/mL, and then samples were harvested for enzyme-linked immunosorbent assay (ELISA) and qPCR analysis. Brefeldin A was purchased from Invitrogen (Carlsbad, CA) and diluted according to the instructions. Disulfiram purchased from Sigma (USA) was dissolved in dimethyl sulfoxide (DMSO). The final concentration used was 0.5 μM.

### PI staining.

293T cells were transfected with plasmids for 24 h and then washed with phosphate-buffered saline (PBS). The cells were stained with 4 μM PI (Sigma-Aldrich, USA) for 10 min at 37°C and visualized using an Evos FL Auto2 fluorescence microscope.

### Cell cytotoxicity and viability.

The cell death and viability were analyzed by the CytoTox 96 nonradioactive cytotoxicity assay and CellTiter-Glo luminescent cell viability assay (Promega, WI) according to the manufacturer’s instructions.

### RNA interference.

siRNAs against IFNAR1 and a nontargeting control were purchased from GenePharma (Shanghai, China), with the following sequence: GCCUGGAUGUCAAUAUGUUTT. ST cells were seeded in 48-well plates. When cells were grown to 80% confluence, siRNAs were transfected using Lipofectamine 2000 transfection reagent (Invitrogen, Carlsbad, CA). After 24 h of transfection, cells were treated with cell supernatant and collected after 24 h for qPCR analyses.

### SDS-PAGE and Western blot analysis.

ST cells were infected with TGEV or transfected with plasmids in 6-well plates; each well of a 6-well plate was lysed with 100 μL of NP-40 buffer (Beyotime, China, Shanghai) containing 0.1% mM phenylmethylsulfonyl fluoride (PMSF) protease inhibitor cocktail (Roche Molecular Biochemicals) for 30 min in ice. After centrifugation for 12,000 × *g* for 10 min at 4°C, SDS was added to the supernatant and boiled for 10 min. Cell lysates were electrophoresed on SDS-PAGE gels, transferred to nitrocellulose filter (NC) membranes (Millipore, Billerica, MA, USA), then blocked with 5% nonfat dry milk in TBST (20 mM Tris [pH 7.4], 150 mM NaCl, 0.1% Tween 20) for 2 h at room temperature, and incubated with the primary antibodies at 4°C overnight. After three washings with TBST, the membrane was incubated with the corresponding IRDye800-conjugated anti-mouse IgG (Invitrogen, Carlsbad, CA) in PBS for 1 h at room temperature. After three washings with TBST, the membrane was scanned and analyzed in an Odyssey infrared imaging system (Li-Cor Biosciences).

### Generation of stable cell lines.

Exon 2 and part of exon 3 of *Gsdmd* were targeted by three guide RNAs (gRNAs) using the website of the Zhang lab (https://zlab.bio/guide-design-resources). Guide 1 (AGGTCAACACATGTGTAGCGGGG), guide 2 (GGGTGGTCAAGAGCGTGGTCCGG), and guide 3 (GAACGTGTGCACGCTACGAGTGG) were connected with pX330-U6-Chimeric_BB-CBh-hSpCas9 plasmid, and then 4 μg of gRNA-expressing pX330 plasmid and 0.4 μg of pEGFP-C2 vector were cotransfected into ST cells. Two days later, green fluorescent protein (GFP)-positive cells were sorted into single clones into the 96-well plate by flow cytometry using the Beckman Coulter MoFlo XDP cell sorter. Single clones were screened by sequencing of the PCR fragments using GSDMD-text-F (5′-TGGCCACGCAGGATCGCTTTGAA-3′) and GSDMD-text-R (5′-GCGGGCCCGTCTGACTGGAACTT-3′) PCR primers and Western blot analysis.

### Virus titration.

Wild-type ST cells, *Gsdmd*^−/−^ ST cells, and plasmid-transfected ST cells were grown in 6-well plates and then infected with TGEV or PDCoV at an MOI of 1. Cell supernatants were collected at different time points, and the cells were used for RNA isolation and indirect immunofluorescence assay (IFA) analysis. The cell supernatants were serially 10-fold diluted from 10^−1^ to 10^−10^ and then added to confluent ST cells in 96-well plates. Virus titers were calculated using the Reed-Muench method after 72 h of infection.

### Immunofluorescence.

Cell samples collected as described previously were washed with PBS two times, fixed with 4% paraformaldehyde for 30 min at room temperature, and then permeabilized with 0.1% Triton X-100 for 10 min at room temperature. PBS containing 5% serum and nonfat dry milk was used as blocking buffer overnight at 4°C, incubated with monoclonal antibodies against TGEV N protein (1:1,000), PDCoV S protein (1:1,000), or anti-HA antibody (1:5,000) for 2 h at 37°C. After three washings with PBST (PBS with 0.05% Tween 20), samples were stained for 1 h with Alexa Fluor 546 goat anti-mouse IgG antibody or Alexa Fluor 488 goat anti-rabbit IgG antibody (1:500; Thermo Fisher Scientific, USA) at 37°C. The nucleus was stained with 4′,6-diamidino-2-phenylindole (DAPI; 1:100; Sigma, USA) and then visualized using an Evos FL Auto2 fluorescence microscope.

### ELISA.

IFN-β and IL-6 sandwich ELISAs were performed using an ELISA kit purchased from Bio-Swamp (Wuhan, China) according to the instruction manual.

### Statistical analysis.

For nonspecial cases, all the results in a graph are displayed as three standard errors of the means (SEMs) from independent experiments and were analyzed by GraphPad Prism (GraphPad software). The two-tailed Student *t* test was used to compare the two data groups, and *P* values of <0.05 were considered significant.

## References

[1] WHO. 2021. WHO coronavirus disease (COVID-2019) situation reports. https://www.who.int/emergencies/diseases/novel-coronavirus-2019/situation-reports.

[2] Cui J, Li F, Shi ZL. 2019. Origin and evolution of pathogenic coronaviruses. Nat Rev Microbiol 17:181–192. doi:10.1038/s41579-018-0118-9. 30531947 PMC7097006

[3] de Wit E, van Doremalen N, Falzarano D, Munster VJ. 2016. SARS and MERS: recent insights into emerging coronaviruses. Nat Rev Microbiol 14:523–534. doi:10.1038/nrmicro.2016.81. 27344959 PMC7097822

[4] Wang Q, Vlasova AN, Kenney SP, Saif LJ. 2019. Emerging and re-emerging coronaviruses in pigs. Curr Opin Virol 34:39–49. doi:10.1016/j.coviro.2018.12.001. 30654269 PMC7102852

[5] Li W, Hulswit RJG, Kenney SP, Widjaja I, Jung K, Alhamo MA, van Dieren B, van Kuppeveld FJM, Saif LJ, Bosch BJ. 2018. Broad receptor engagement of an emerging global coronavirus may potentiate its diverse cross-species transmissibility. Proc Natl Acad Sci U S A 115:E5135–E5143. doi:10.1073/pnas.1802879115. 29760102 PMC5984533

[6] Zhu X, Liu S, Wang X, Luo Z, Shi Y, Wang D, Peng G, Chen H, Fang L, Xiao S. 2018. Contribution of porcine aminopeptidase N to porcine deltacoronavirus infection. Emerg Microbes Infect 7:65. doi:10.1038/s41426-018-0068-3. 29636467 PMC5893578

[7] Tusell SM, Schittone SA, Holmes KV. 2007. Mutational analysis of aminopeptidase N, a receptor for several group 1 coronaviruses, identifies key determinants of viral host range. J Virol 81:1261–1273. doi:10.1128/JVI.01510-06. 17093189 PMC1797531

[8] Vora SM, Lieberman J, Wu H. 2021. Inflammasome activation at the crux of severe COVID-19. Nat Rev Immunol 21:694–703. doi:10.1038/s41577-021-00588-x. 34373622 PMC8351223

[9] Israelow B, Song E, Mao T, Lu P, Meir A, Liu F, Alfajaro MM, Wei J, Dong H, Homer RJ, Ring A, Wilen CB, Iwasaki A. 2020. Mouse model of SARS-CoV-2 reveals inflammatory role of type I interferon signaling. J Exp Med 217:e20201241. doi:10.1084/jem.20201241. 32750141 PMC7401025

[10] Frank D, Vince JE. 2019. Pyroptosis versus necroptosis: similarities, differences, and crosstalk. Cell Death Differ 26:99–114. doi:10.1038/s41418-018-0212-6. 30341423 PMC6294779

[11] Bergsbaken T, Fink SL, Cookson BT. 2009. Pyroptosis: host cell death and inflammation. Nat Rev Microbiol 7:99–109. doi:10.1038/nrmicro2070. 19148178 PMC2910423

[12] Cookson BT, Brennan MA. 2001. Pro-inflammatory programmed cell death. Trends Microbiol 9:113–114. doi:10.1016/s0966-842x(00)01936-3. 11303500

[13] Shi J, Zhao Y, Wang K, Shi X, Wang Y, Huang H, Zhuang Y, Cai T, Wang F, Shao F. 2015. Cleavage of GSDMD by inflammatory caspases determines pyroptotic cell death. Nature 526:660–665. doi:10.1038/nature15514. 26375003

[14] Kuang S, Zheng J, Yang H, Li S, Duan S, Shen Y, Ji C, Gan J, Xu XW, Li J. 2017. Structure insight of GSDMD reveals the basis of GSDMD autoinhibition in cell pyroptosis. Proc Natl Acad Sci U S A 114:10642–10647. doi:10.1073/pnas.1708194114. 28928145 PMC5635896

[15] Liu Z, Wang C, Rathkey JK, Yang J, Dubyak GR, Abbott DW, Xiao TS. 2018. Structures of the gasdermin D C-terminal domains reveal mechanisms of autoinhibition. Structure 26:778–784.e773. doi:10.1016/j.str.2018.03.002. 29576317 PMC5932255

[16] Wang K, Sun Q, Zhong X, Zeng M, Zeng H, Shi X, Li Z, Wang Y, Zhao Q, Shao F, Ding J. 2020. Structural mechanism for GSDMD targeting by autoprocessed caspases in pyroptosis. Cell 180:941–955.e920. doi:10.1016/j.cell.2020.02.002. 32109412

[17] Ding J, Wang K, Liu W, She Y, Sun Q, Shi J, Sun H, Wang DC, Shao F. 2016. Pore-forming activity and structural autoinhibition of the gasdermin family. Nature 535:111–116. doi:10.1038/nature18590. 27281216

[18] Liu Z, Wang C, Yang J, Zhou B, Yang R, Ramachandran R, Abbott DW, Xiao TS. 2019. Crystal structures of the full-length murine and human gasdermin D reveal mechanisms of autoinhibition, lipid binding, and oligomerization. Immunity 51:43–49.e44. doi:10.1016/j.immuni.2019.04.017. 31097341 PMC6640092

[19] Ruan J. 2019. Structural insight of gasdermin family driving pyroptotic cell death. Adv Exp Med Biol 1172:189–205. doi:10.1007/978-981-13-9367-9_9. 31628657

[20] Sborgi L, Ruhl S, Mulvihill E, Pipercevic J, Heilig R, Stahlberg H, Farady CJ, Muller DJ, Broz P, Hiller S. 2016. GSDMD membrane pore formation constitutes the mechanism of pyroptotic cell death. EMBO J 35:1766–1778. doi:10.15252/embj.201694696. 27418190 PMC5010048

[21] Aglietti RA, Estevez A, Gupta A, Ramirez MG, Liu PS, Kayagaki N, Ciferri C, Dixit VM, Dueber EC. 2016. GsdmD p30 elicited by caspase-11 during pyroptosis forms pores in membranes. Proc Natl Acad Sci U S A 113:7858–7863. doi:10.1073/pnas.1607769113. 27339137 PMC4948338

[22] de Vasconcelos NM, Van Opdenbosch N, Van Gorp H, Parthoens E, Lamkanfi M. 2019. Single-cell analysis of pyroptosis dynamics reveals conserved GSDMD-mediated subcellular events that precede plasma membrane rupture. Cell Death Differ 26:146–161. doi:10.1038/s41418-018-0106-7. 29666477 PMC6294780

[23] Junqueira C, Crespo A, Ranjbar S, Lewandrowski M, Ingber J, de Lacerda LB, Parry B, Ravid S, Clark S, Ho F, Vora SM, Leger V, Beakes C, Margolin J, Russell N, Kays K, Gehrke L, Adhikari UD, Henderson L, Janssen E, Kwon D, Sander C, Abraham J, Filbin M, Goldberg MB, Wu H, Mehta G, Bell S, Goldfeld AE, Lieberman J. 11 August 2021. SARS-CoV-2 infects blood monocytes to activate NLRP3 and AIM2 inflammasomes, pyroptosis and cytokine release. Res Sq doi:10.21203/rs.3.rs-153628/v1.

[24] Yap JKY, Moriyama M, Iwasaki A. 2020. Inflammasomes and pyroptosis as therapeutic targets for COVID-19. J Immunol 205:307–312. doi:10.4049/jimmunol.2000513. 32493814 PMC7343621

[25] Zheng M, Williams EP, Malireddi RKS, Karki R, Banoth B, Burton A, Webby R, Channappanavar R, Jonsson CB, Kanneganti TD. 2020. Impaired NLRP3 inflammasome activation/pyroptosis leads to robust inflammatory cell death via caspase-8/RIPK3 during coronavirus infection. J Biol Chem 295:14040–14052. doi:10.1074/jbc.RA120.015036. 32763970 PMC7549031

[26] Wei G, Luo S, Wu W, Hu J, Zhou R. 2021. Activation of interleukin-1beta release and pyroptosis by transmissible gastroenteritis virus is dependent on the NOD-like receptor protein 3 inflammasome in porcine intestinal epithelial cell line. Viral Immunol 34:401–409. doi:10.1089/vim.2020.0227. 33973805

[27] Kayagaki N, Warming S, Lamkanfi M, Vande Walle L, Louie S, Dong J, Newton K, Qu Y, Liu J, Heldens S, Zhang J, Lee WP, Roose-Girma M, Dixit VM. 2011. Non-canonical inflammasome activation targets caspase-11. Nature 479:117–121. doi:10.1038/nature10558. 22002608

[28] Sarhan J, Liu BC, Muendlein HI, Li P, Nilson R, Tang AY, Rongvaux A, Bunnell SC, Shao F, Green DR, Poltorak A. 2018. Caspase-8 induces cleavage of gasdermin D to elicit pyroptosis during Yersinia infection. Proc Natl Acad Sci U S A 115:E10888–E10897. doi:10.1073/pnas.1809548115. 30381458 PMC6243247

[29] Rühl S, Broz P. 2015. Caspase-11 activates a canonical NLRP3 inflammasome by promoting K(+) efflux. Eur J Immunol 45:2927–2936. doi:10.1002/eji.201545772. 26173909

[30] Xia S, Zhang Z, Magupalli VG, Pablo JL, Dong Y, Vora SM, Wang L, Fu TM, Jacobson MP, Greka A, Lieberman J, Ruan J, Wu H. 2021. Gasdermin D pore structure reveals preferential release of mature interleukin-1. Nature 593:607–611. doi:10.1038/s41586-021-03478-3. 33883744 PMC8588876

[31] Ferreira AC, Soares VC, de Azevedo-Quintanilha IG, Dias S, Fintelman-Rodrigues N, Sacramento CQ, Mattos M, de Freitas CS, Temerozo JR, Teixeira L, Damaceno Hottz E, Barreto EA, Pao CRR, Palhinha L, Miranda M, Bou-Habib DC, Bozza FA, Bozza PT, Souza TML. 2021. SARS-CoV-2 engages inflammasome and pyroptosis in human primary monocytes. Cell Death Discov 7:43. doi:10.1038/s41420-021-00428-w. 33649297 PMC7919254

[32] Hoffmann HH, Schneider WM, Rice CM. 2015. Interferons and viruses: an evolutionary arms race of molecular interactions. Trends Immunol 36:124–138. doi:10.1016/j.it.2015.01.004. 25704559 PMC4384471

[33] Schreiber G, Piehler J. 2015. The molecular basis for functional plasticity in type I interferon signaling. Trends Immunol 36:139–149. doi:10.1016/j.it.2015.01.002. 25687684

[34] Collins SE, Mossman KL. 2014. Danger, diversity and priming in innate antiviral immunity. Cytokine Growth Factor Rev 25:525–531. doi:10.1016/j.cytogfr.2014.07.002. 25081316

[35] Gu Y, Kuida K, Tsutsui H, Ku G, Hsiao K, Fleming MA, Hayashi N, Higashino K, Okamura H, Nakanishi K, Kurimoto M, Tanimoto T, Flavell RA, Sato V, Harding MW, Livingston DJ, Su MS. 1997. Activation of interferon-gamma inducing factor mediated by interleukin-1beta converting enzyme. Science 275:206–209. doi:10.1126/science.275.5297.206. 8999548

[36] Coccia EM, Battistini A. 2015. Early IFN type I response: learning from microbial evasion strategies. Semin Immunol 27:85–101. doi:10.1016/j.smim.2015.03.005. 25869307 PMC7129383

[37] Schneider WM, Chevillotte MD, Rice CM. 2014. Interferon-stimulated genes: a complex web of host defenses. Annu Rev Immunol 32:513–545. doi:10.1146/annurev-immunol-032713-120231. 24555472 PMC4313732

[38] He WT, Wan H, Hu L, Chen P, Wang X, Huang Z, Yang ZH, Zhong CQ, Han J. 2015. Gasdermin D is an executor of pyroptosis and required for interleukin-1β secretion. Cell Res 25:1285–1298. doi:10.1038/cr.2015.139. 26611636 PMC4670995

[39] Burke TP, Engstrom P, Chavez RA, Fonbuena JA, Vance RE, Welch MD. 2020. Inflammasome-mediated antagonism of type I interferon enhances Rickettsia pathogenesis. Nat Microbiol 5:688–696. doi:10.1038/s41564-020-0673-5. 32123346 PMC7239376

[40] Fan S, Yuan J, Deng S, Chen Y, Xie B, Wu K, Zhu M, Xu H, Huang Y, Yang J, Zhang Y, Chen J, Zhao M. 2018. Activation of interleukin-1beta release by the classical swine fever virus is dependent on the NLRP3 inflammasome, which affects virus growth in monocytes. Front Cell Infect Microbiol 8:225. doi:10.3389/fcimb.2018.00225. 30013955 PMC6036178

[41] La Bonnardiere C, Laude H. 1981. High interferon titer in newborn pig intestine during experimentally induced viral enteritis. Infect Immun 32:28–31. doi:10.1128/iai.32.1.28-31.1981. 6163724 PMC350581

[42] Riffault S, Carrat C, van Reeth K, Pensaert M, Charley B. 2001. Interferon-alpha-producing cells are localized in gut-associated lymphoid tissues in transmissible gastroenteritis virus (TGEV) infected piglets. Vet Res 32:71–79. doi:10.1051/vetres:2001111. 11254179

[43] Yin L, Chen J, Li L, Guo S, Xue M, Zhang J, Liu X, Feng L, Liu P. 2020. Aminopeptidase N expression, not interferon responses, determines the intestinal segmental tropism of porcine deltacoronavirus. J Virol 94:e00480-20. doi:10.1128/JVI.00480-20. 32376622 PMC7343211

[44] Ma C, Yang D, Wang B, Wu C, Wu Y, Li S, Liu X, Lassen K, Dai L, Yang S. 2020. Gasdermin D in macrophages restrains colitis by controlling cGAS-mediated inflammation. Sci Adv 6:eaaz6717. doi:10.1126/sciadv.aaz6717. 32671214 PMC7314554

[45] Banerjee I, Behl B, Mendonca M, Shrivastava G, Russo AJ, Menoret A, Ghosh A, Vella AT, Vanaja SK, Sarkar SN, Fitzgerald KA, Rathinam VAK. 2018. Gasdermin D restrains type I interferon response to cytosolic DNA by disrupting ionic homeostasis. Immunity 49:413–426.e415. doi:10.1016/j.immuni.2018.07.006. 30170814 PMC6347470

[46] Liu X, Zhang Z, Ruan J, Pan Y, Magupalli VG, Wu H, Lieberman J. 2016. Inflammasome-activated gasdermin D causes pyroptosis by forming membrane pores. Nature 535:153–158. doi:10.1038/nature18629. 27383986 PMC5539988

[47] Chen X, He WT, Hu L, Li J, Fang Y, Wang X, Xu X, Wang Z, Huang K, Han J. 2016. Pyroptosis is driven by non-selective gasdermin-D pore and its morphology is different from MLKL channel-mediated necroptosis. Cell Res 26:1007–1020. doi:10.1038/cr.2016.100. 27573174 PMC5034106

[48] Liu Z, Wang C, Yang J, Chen Y, Zhou B, Abbott DW, Xiao TS. 2020. Caspase-1 engages full-length gasdermin D through two distinct interfaces that mediate caspase recruitment and substrate cleavage. Immunity 53:106–114.e105. doi:10.1016/j.immuni.2020.06.007. 32553275 PMC7382298

[49] Kayagaki N, Stowe IB, Lee BL, O’Rourke K, Anderson K, Warming S, Cuellar T, Haley B, Roose-Girma M, Phung QT, Liu PS, Lill JR, Li H, Wu J, Kummerfeld S, Zhang J, Lee WP, Snipas SJ, Salvesen GS, Morris LX, Fitzgerald L, Zhang Y, Bertram EM, Goodnow CC, Dixit VM. 2015. Caspase-11 cleaves gasdermin D for non-canonical inflammasome signalling. Nature 526:666–671. doi:10.1038/nature15541. 26375259

[50] Ma Y, Wang C, Xue M, Fu F, Zhang X, Li L, Yin L, Xu W, Feng L, Liu P. 2018. The coronavirus transmissible gastroenteritis virus evades the type I interferon response through IRE1alpha-mediated manipulation of the microRNA miR-30a-5p/SOCS1/3 axis. J Virol 92:e00728-18. doi:10.1128/JVI.00728-18. 30185587 PMC6206482

[51] Park A, Iwasaki A. 2020. Type I and type III interferons—induction, signaling, evasion, and application to combat COVID-19. Cell Host Microbe 27:870–878. doi:10.1016/j.chom.2020.05.008. 32464097 PMC7255347

[52] Rubartelli A, Cozzolino F, Talio M, Sitia R. 1990. A novel secretory pathway for interleukin-1 beta, a protein lacking a signal sequence. EMBO J 9:1503–1510. doi:10.1002/j.1460-2075.1990.tb08268.x. 2328723 PMC551842

[53] Lieberman J, Wu H, Kagan JC. 2019. Gasdermin D activity in inflammation and host defense. Sci Immunol 4:eaav1447. doi:10.1126/sciimmunol.aav1447. 31492708 PMC7004224

[54] Mulvihill E, Sborgi L, Mari SA, Pfreundschuh M, Hiller S, Muller DJ. 2018. Mechanism of membrane pore formation by human gasdermin-D. EMBO J 37:e98321. doi:10.15252/embj.201798321. 29898893 PMC6043855

[55] Man SM, Karki R, Kanneganti TD. 2017. Molecular mechanisms and functions of pyroptosis, inflammatory caspases and inflammasomes in infectious diseases. Immunol Rev 277:61–75. doi:10.1111/imr.12534. 28462526 PMC5416822

[56] Xu Z, Zhong H, Huang S, Zhou Q, Du Y, Chen L, Xue C, Cao Y. 2019. Porcine deltacoronavirus induces TLR3, IL-12, IFN-alpha, IFN-beta and PKR mRNA expression in infected Peyer’s patches in vivo. Vet Microbiol 228:226–233. doi:10.1016/j.vetmic.2018.12.012. 30593372 PMC7117130

[57] Dubois H, Sorgeloos F, Sarvestani ST, Martens L, Saeys Y, Mackenzie JM, Lamkanfi M, van Loo G, Goodfellow I, Wullaert A. 2019. Nlrp3 inflammasome activation and gasdermin D-driven pyroptosis are immunopathogenic upon gastrointestinal norovirus infection. PLoS Pathog 15:e1007709. doi:10.1371/journal.ppat.1007709. 31017981 PMC6502405

[58] Orning P, Lien E, Fitzgerald KA. 2019. Gasdermins and their role in immunity and inflammation. J Exp Med 216:2453–2465. doi:10.1084/jem.20190545. 31548300 PMC6829603

[59] Shi J, Gao W, Shao F. 2017. Pyroptosis: gasdermin-mediated programmed necrotic cell death. Trends Biochem Sci 42:245–254. doi:10.1016/j.tibs.2016.10.004. 27932073

[60] Zhang J, Wu H, Yao X, Zhang D, Zhou Y, Fu B, Wang W, Li H, Wang Z, Hu Z, Ren Y, Sun R, Tian Z, Bian X, Wei H. 2021. Pyroptotic macrophages stimulate the SARS-CoV-2-associated cytokine storm. Cell Mol Immunol 18:1305–1307. doi:10.1038/s41423-021-00665-0. 33742186 PMC7976727

[61] Zhu S, Ding S, Wang P, Wei Z, Pan W, Palm NW, Yang Y, Yu H, Li HB, Wang G, Lei X, de Zoete MR, Zhao J, Zheng Y, Chen H, Zhao Y, Jurado KA, Feng N, Shan L, Kluger Y, Lu J, Abraham C, Fikrig E, Greenberg HB, Flavell RA. 2017. Nlrp9b inflammasome restricts rotavirus infection in intestinal epithelial cells. Nature 546:667–670. doi:10.1038/nature22967. 28636595 PMC5787375

[62] Lei X, Zhang Z, Xiao X, Qi J, He B, Wang J. 2017. Enterovirus 71 inhibits pyroptosis through cleavage of gasdermin D. J Virol 91:e01069-17. doi:10.1128/JVI.01069-17. 28679757 PMC5571240

[63] Evavold CL, Ruan J, Tan Y, Xia S, Wu H, Kagan JC. 2018. The pore-forming protein gasdermin D regulates interleukin-1 secretion from living macrophages. Immunity 48:35–44.e36. doi:10.1016/j.immuni.2017.11.013. 29195811 PMC5773350

[64] Murray RZ, Stow JL. 2014. Cytokine secretion in macrophages: SNAREs, Rabs, and membrane trafficking. Front Immunol 5:538. doi:10.3389/fimmu.2014.00538. 25386181 PMC4209870

[65] Ivashkiv LB, Donlin LT. 2014. Regulation of type I interferon responses. Nat Rev Immunol 14:36–49. doi:10.1038/nri3581. 24362405 PMC4084561

[66] Stanley AC, Lacy P. 2010. Pathways for cytokine secretion. Physiology (Bethesda) 25:218–229. doi:10.1152/physiol.00017.2010. 20699468

[67] Ma J, Zhu F, Zhao M, Shao F, Yu D, Ma J, Zhang X, Li Q, Qian Y, Zhang Y, Jiang D, Wang S, Xia P. 2021. SARS-CoV-2 nucleocapsid suppresses host pyroptosis by blocking gasdermin D cleavage. EMBO J e108249. doi:10.15252/embj.2021108249. 34296442 PMC8420271

