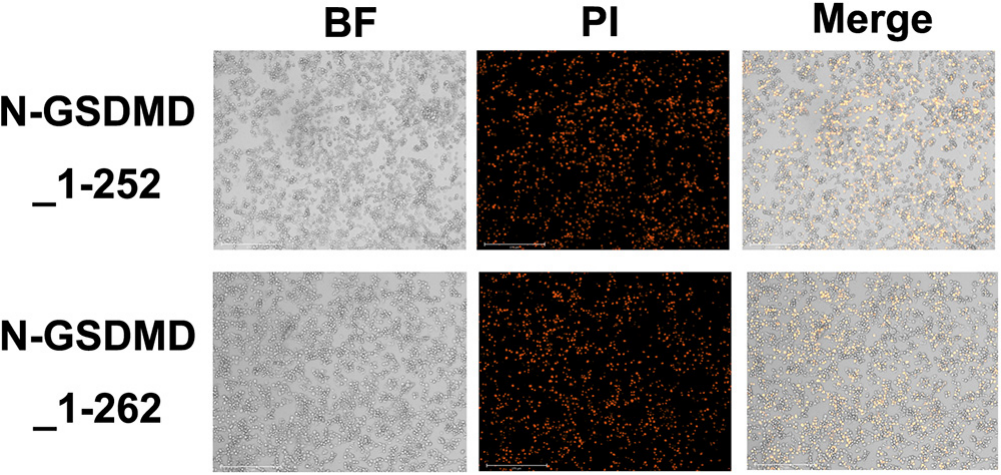# Correction for Zhao et al., “Gasdermin D Inhibits Coronavirus Infection by Promoting the Noncanonical Secretion of Beta Interferon”

**DOI:** 10.1128/mbio.02586-22

**Published:** 2022-10-03

**Authors:** Liyuan Zhao, Liang Li, Mei Xue, Xiang Liu, Chengfan Jiang, Wenzhe Wang, Lijie Tang, Li Feng, Pinghuang Liu

**Affiliations:** a State Key Laboratory of Veterinary Biotechnology, Harbin Veterinary Research Institute, Chinese Academy of Agricultural Sciences, Harbin, China; b College of Veterinary Medicine, China Agricultural University, Beijing, China; c College of Veterinary Medicine, Northeast Agricultural University, Harbin, China

## AUTHOR CORRECTION

Volume 13, no. 1, e03600-21, 2022, https://doi.org/10.1128/mbio.03600-21. In Fig. 2I, there are partial duplications in the N-GSDMD_1-242 and N-GSDMD_1-252 panels and the N-GSDMD_1-252 and N-GSDMD_1-262 panels[Fig F2].

Due to the large number of original pictures, when processing the data, the authors inadvertently placed the results of different angles of N-GSDMD_1-242 to the positions of N-GSDMD_1-252 and N-GSDMD_1-262 by mistake. Figure 2I has been updated with the correct images, as shown below. The authors apologize for the error and state that the error does not change the scientific conclusions of the article in any way.

**Figure F2:**